# Tissue Optical Clearing: State of the Art and Prospects

**DOI:** 10.3390/diagnostics12071534

**Published:** 2022-06-23

**Authors:** Elina A. Genina

**Affiliations:** 1 Optics and Biophotonics Department, Saratov State University, 83 Astrakhanskaya Str., 410012 Saratov, Russia; eagenina@yandex.ru; 2 Laboratory of Laser Molecular Imaging and Machine Learning, Tomsk State University, 36 Lenin’s Av., 634050 Tomsk, Russia

The term “tissue optical clearing” (TOC) came into use at the end of the 20th century and is associated with the development of methods for controlling tissue scattering properties using the refractive index matching effect. However, the first mention of increasing the transparency of biological tissues using immersion agents can be attributed to the work of Spalteholz in 1914 [1], in which an organic solvent-based technique was applied to tissue samples in vitro. The next step was made by Barer et al., in 1955 [2], who proposed the optical clearing of cell suspensions by means of protein solution with the same refractive index as the cell cytoplasm. The use of the TOC approach for an increase in eye sclera transparency was first applied by a scientific group with Tuchin in 1987 [3]. The authors demonstrated, theoretically and experimentally, the possibility of effective control of the scattering properties of sclera due to verografin solution. The works published from the mid-1990s by a number of leading scientific groups opened up a new direction in the study of TOC [4,5,6]. The rapid evolution of optical diagnostic and research methods induced comprehensive study of the mechanisms of TOC, a search for new approaches and optical clearing agents (OCAs). The development of understanding of the features and mechanisms of the phenomenon of TOC in vitro, ex vivo and in vivo demonstrated the method’s capabilities to increase the probing depth or the image contrast of optical devices, as reflected in reviews [7,8,9,10,11]. It was also found that the efficiency of TOC and the diffusion rate of OCAs differ in normal and pathological tissues that can be used as an additional diagnostic marker, for example, for diabetes [12] and cancer [13].

The development of fluorescence microscopy provided the possibility of obtaining a 3D reconstruction of the tissue anatomy and stimulated the development of innovative protocols of TOC and a new generation of OCAs for fixed samples. These protocols made it possible to almost completely overcome light scattering in tissues, organs or small animals and make them optically transparent materials. The FocusClear™ solution was one of the first specially designed OCAs, which was used by Chiang et al. in 2001 for the clearing of fixed insect organs with immunohistochemical labeling [14]. An alternative approach based on hyperhydration of tissue samples using the Sca*l*e technique was developed by Hama et al. in 2011 [15]. In 2013, hydrogel-tissue-hybridization called CLARITY was suggested by Chung et al. [16]. Detailed reviews are presented in Refs. [17,18,19] and book chapters [20].

Most soft tissues are characterized by a heterogeneous composition, including relatively large structures in the form of cells, fibers, lipid droplets and smaller components, such as cell organelles and phospholipid membranes. All of them are distributed in a water-based background matrix. Thus, tissues have a wide range of refractive indices, from ~1.34 for interstitial fluid and cytoplasm to >1.44 for proteins and lipids. The inorganic matrix of bones contains calcium hydroxyapatite with a refractive index >1.6. Refractive index mismatch of these components and the disordered packing of fibers in tissues are sources of strong light scattering. The main purpose of TOC is to match the refractive indices of the various tissue components using OCAs. However, different approaches reach this goal in different ways.

As a first approximation, the TOC process can be divided into three stages. The exchange flow of free water from the tissue to the OCA and the OCA to the tissue is characterized by different rates. Thus, at the first stage, hyperosmotic OCA causes a predominant loss of water in the tissues (dehydration). At the second stage, the diffusion of OCA in the tissue predominates, and at the third stage, the OCAs interact with the tissue components [21].

Molecular dynamics modeling has shown that the experimentally observed degree of TOC and parameters, such as osmolarity and refractive index, of OCA do not correlate. In Refs. [22,23,24], the hydrogen bond formation between different OCAs (alcohols and sugars) and collagen molecules was simulated. It has been shown that the higher conformational mobility of the low-molecular OCAs provides better adaptation of the agent to the collagen molecular pocket. OCA molecules partially displace bound water and disrupt the network of hydrogen bonds between collagen fibrils, which leads to swelling of fibrillar proteins at the third stage of TOC [24].

TOC mechanisms and approaches are discussed in detail in Ref. [21]. Briefly, TOC can be divided into several approaches depending on specific scientific problems: (i) simple immersion with the use of hyperosmotic or lipophilic OCAs, as one-component agents, as well as multi-component solutions; (ii) immersion with previous dehydration and delipidation with the use of organic solvents; (iii) hyperhydration with the use of delipidation and partial denaturation of tissue proteins; and (iv) hydrogel embedding with the use of fixation and crosslinking. The first two methods are associated with an increase in the average refractive index of tissue by replacing tissue water with a fluid with a higher refractive index. In contrast, the last two approaches involve removing lipids and reducing the refractive index of tissue samples to ~1.38. In hyperhydration, urea is used as a component for partial denaturation and hydration of hydrophobic regions of proteins. The fourth method allows fixing proteins and nucleic acids in their physiological locations by covalently linking the molecules to an acrylic-based hydrogel. Thus, the tissue samples can then be successfully cleared with OCAs, achieving high transparency. Some TOC protocols also allow for tissue decalcification and decolorization [21].

The first approach was successfully used in the development of in-vivo TOC. In these cases, TOC protocols should also provide for increasing the permeability of tissues and ensuring the safety and reversibility of the procedure.

In the last decade, a great variety of TOC methods based on different approaches was developed to achieve complete transparency of fixed tissue for fluorescence microscopy. Specially optimized multi-component solutions with innovative protocols, such as ACT-PRESTO, CLARITY, CRISTAL, CUBIC, DISCO, eFLASH, FlyClear, FRUIT, MOVIE, PEGASOS, Scale, SHANEL, SWITCH and many others, were suggested for application in visualization techniques [17,18,19,20,25]. They provided the effective clearing of tissues and whole organs, including bones. For example, vDISCO allowed 3D visualization of whole-body neuronal connectivity, meningeal lymphatic vessels and immune cells through the intact skull and vertebra in naive animals and trauma models [26]. An approach combining CLARITY with double-photon microscopy was proposed for the visualization of gray matter in the intact brain [27]. The CUBIC protocol was shown to be effective for an improvement in confocal imaging of mouse heart sections [28] and 3D vasculature images of a whole mouse heart [29]. A combination of CUBIC and CLARITY was used to evaluate the spatial distribution and phenotype of fibroblasts in mice left ventricles in a postmyocardial infarction [30]. A high-resolution whole mouse brain atlas was achieved by using CUBIC-X [31]. In order to better preserve tissue architecture and fluorescence, an alternative hydrogel-based method, named SHIELD, was used to evaluate the synaptic architecture of virally labeled murine neurons at single-cell resolution [32]. A hydrogel-based TOC method, MYOCLEAR, was developed for the labeling and detection of all neuromuscular junctions and diaphragm muscles in mice [33]. SHANEL protocol [34] was applied to large lipid-rich samples, such as human and porcine tissues, for clearing and decolorization.

TOC in vivo and ex vivo is realized using biocompatible OCAs separately or in combination with chemical and physical actions for enhancement of tissue permeability. In this case, biologically safe solvents in small concentrations can be included into the content of OCAs [35,36,37]. The use of DMSO, sonophoresis, laser irradiation, microdermabrasion, microperforation and other enhancers of OCA penetration in tissues are described in Refs. [38,39,40,41,42]. It is important for the development of these approaches to provide tissue viability. After the removal of OCAs, the tissue structure, functions and optical properties should be totally restored.

TOC protocols developed for skull clearing in vivo (SOCS, USOCA, SOCW, etc.) allowed for the visualization of blood vessels and brain tissues in mice without removing the cranial bone [36,43,44].

Despite significant progress in TOC, several important challenges remain. The variety of existing TOC methods and the constant work on the creation of new approaches indicate the impossibility of creating a universal protocol. The choice of the appropriate TOC protocol depends on tissue size and type, type of fluorescence, importance of tissue shrinkage and clearing time [17].

Until now, for many TOC approaches, the choice of OCA components and parameters for the additional enhancement of tissue permeability has been random and is not justified by the results of preliminary systematic studies. However, it is known that changes in the optical, weight and geometric parameters of tissue under the action of immersion fluids have significant concentration dependence [45]. Further, it was found that the efficiency of the subsequent clearing of fixed samples depends on the pH of fixation [46]. Taking into account such data would help to increase the efficiency of the development of new optical clearing methods. Molecular dynamics modeling can be useful to predict optimal OCAs.

Another important problem is the absorption of light by endogenous pigments, such as hemoglobin, myoglobin and melanin, which limits both the excitation light entering the tissue and the fluorescent radiation returning to the detector [17,21]. This requires the inclusion of additional bleaching components to TOC protocols [32,47].

Many organic solvents used cause a substantial dehydration and, thus, tissue shrinkage, as well as the quenching of fluorescent protein emissions [18]. To overcome these problems, it is necessary to complicate and prolong TOC protocols. Thus, sample preparation can take more than a month in some cases.

An example of the development of OCA based on chemical screening allowed expanding the palette of CUBIC approaches and the use of electrophoresis for delipidation and decolorization speeded up the procedure from a month to 2 weeks [48]. The MACS protocol based on a new hyperhydration reagent—M-xylylenediamine (MXDA)—possessed superior hyperhydration ability and showed much faster clearing than the Sca*l*e and CUBIC series methods [49].

Further refinements of the TOC methods contribute to whole-cell profiling of the human body. Therefore, the SHANEL method reported by Zhao at al. was applied to clear the entire human brain and kidney [34].

In in-vivo studies, a significant reduction in treatment time (no more than tens of minutes) and the use of only biologically compatible agents should be provided. It should also take into account the physiological response of living tissue to the action of the agent and its redistribution with a possible rapid exit from the area of interest. Therefore, the use of laser ablation and microporation in the stratum corneum ex vivo showed an increase in the penetration of OCA into the dermis; however, these treatments caused skin erythema and edema when applied in vivo, which increased both the absorption and scattering of light [40,50]. On the contrary, OCA in combination with sonophoresis caused rapid and safe optical clearing of the skin [41].

In parallel with the development of new TOC protocols, new microscopy technologies using clearing-specific objectives will follow. In addition to improved data collection approaches, the analysis and comparison of massive datasets is a relevant issue. Converting all human cells into digitized data is one of the ultimate goals of TOC technology [17,18,19,20,25]. Deep machine learning can be useful for getting statistically robust conclusions.

In the near future, the development of new methods will continue along with preclinical validation and the transfer of TOC approaches from the laboratory to clinical practice.
